# Rejoinder: “Adaptive versus fixed designs in confirmatory clinical trials: Centering the choice on ethics”

**DOI:** 10.1177/17407745261469874

**Published:** 2026-08-03

**Authors:** Stef Baas, Lukas Pin, David S. Robertson, Alex John London, Mark Sheehan, Sofía S. Villar

**Affiliations:** 1 MRC Biostatistics Unit, University of Cambridge, UK; 2 Philosophy Department, Carnegie Mellon University, Pittsburgh, PA, USA; 3 Ethox Centre, Oxford Population Health, University of Oxford, UK; 4 NIHR Oxford Biomedical Research Centre, Oxford University Hospital Trust, UK

We thank Begg, Senn, and Wittes for their thoughtful and diverse commentaries^1–3^ underscoring our central thesis: that clinical trial designs are not merely technical exercises but involve profound ethical assessments. However, their responses also illustrate the persistent barriers our paper seeks to address, namely, linguistic fragmentation, disagreements about strategies for managing design uncertainty, and the need for deeper engagement with ethical frameworks.

## Addressing the “linguistic quibble”

A striking theme across the commentaries is the lack of consensus on what “adaptive” actually means. Wittes prefers a definition involving changes to the assumed distributional parameters. Senn mainly distinguishes between ratio-altering and non–ratio-altering designs. Begg asserts that group sequential designs (GSDs) are so ubiquitous that “fixed” designs are effectively a “straw man.” However, published empirical evidence indicates that GSDs are still uncommon in practice, for example, a review^4^ from 2016 found 74 trials with a GSD of 54.752 registered phase I–III clinical trials, while another review^5^ furthermore found that only 576 of their 1.772 considered randomized controlled trials (RCTs) published in flagship journals before 2005 used a data monitoring committee or interim analyses during the trial.

This fragmentation is more than a linguistic quibble; it is a pragmatic obstacle. Senn reiterates our point that different parties often find themselves “talking past each other.” To mitigate this, we maintain that a “rational and ethical approach” requires the use of definitions that foster fair assessments by grouping designs on the basis of their scientific and ethically relevant properties. We therefore reiterate our definition: an adaptive design is any trial where design modifications are pre-specified in the protocol and driven by emerging data. This aligns with established regulatory standards from the Food and Drug Administration (FDA)^6^ and European Medicines Agency (EMA),^7^ and directly mirrors the definition proposed in the draft ICH E20 guideline.^8^ Failure to adopt standardized definitions into our common scientific practice is a barrier to identifying and promoting methodologically and ethically sound designs.

Paradoxically, restricting “adaptive” to designs that alter underlying distributional parameter assumptions creates a paradox: a pre-specified response-adaptive algorithm shifting ratios from 1:1:1 to 1:1:0 modifies no distributional assumptions mid-trial, making it “non-adaptive” under Wittes’ criteria. By classifying adaptive designs solely on allocation ratio changes, Senn’s taxonomy risks oversimplifying inferential reality. Classical GSDs are frequently assumed to be inherently unbiased due to their concurrent controls, yet estimation bias can persist in these traditional settings as well.^9^ Furthermore, platform designs can incorporate concurrent controls.^10^ Ultimately, a constant allocation ratio is neither a necessary nor sufficient condition for validity or efficiency, as both depend on using analysis methods tailored to the specific design, rather than rigid taxonomic labels.

Senn rightly observes that planned target effects should reflect clinically meaningful differences dictated by the disease, rather than a drug’s assumed effect. To clarify, our reference to “expected effect sizes” was intended strictly to address scenarios where unrecognized variability impacts sample size calculations, not to advocate for a mid-trial shift in baseline clinical success thresholds.

## Complexity, monitoring, and institutional memory

We appreciate Wittes’ focus on Data Safety Monitoring Boards (DSMBs). While trial complexity requires operational clarity, arguing that adaptive designs impose an overwhelming burden of nuance on the DSMB misinterprets their role. A middle ground must be struck: adaptive designs admittedly introduce unique conceptual complexities for the DSMB. Navigating these nuances requires timely, clear communication from experienced trial statisticians. However, we must not overstate the committee’s operational reach. There remains a strict distinction between ad hoc decisions made by a DSMB in reaction to truly unforeseen external events, and protocol-driven adaptations where their role shifts from reactive decision-making to planned implementation oversight without an expectation to re-evaluate the algorithm’s statistical merits mid-trial.

The DSMB’s mandate remains monitoring patient safety and risk-benefit ratios. Crucially, while the committee must understand how adaptations influence incoming data to correctly monitor trial signals, allowing a DSMB to arbitrarily deviate from a pre-specified algorithm without a clear safety justification actively destroys the trial’s statistical integrity, risking type I error rate inflation and rendering the final data uninterpretable to regulatory agencies.

Begg’s assertion that our advocacy for protocol-defined pre-specification implies a design “set in stone” represents a straw man argument: we do not suggest that an adaptive design can or should anticipate every imaginable trial contingency; truly unforeseen external events will always require the “enlightened judgment” of a DSMB. However, passively accepting known planning uncertainties as an unresolvable reality is a failure of design, not a virtue. If methodologists are acutely aware of these uncertainties, the rational response is to actively build flexibility into the protocol via pre-specified adaptive rules, rather than ignoring them during planning.

While the DSMB remains essential for safeguarding against the genuinely unexpected, relying on a committee’s ad hoc mid-course corrections to salvage a rigidly mis-specified fixed trial is no substitute for a rigorous blueprint. An adaptive design is precisely the mechanism that allows a study to dynamically and efficiently navigate known, anticipated uncertainties *by design*, rather than relying on reactive acceptance.

We note that Begg’s assertion that adaptive designs are treated here as a ‘recent methodological novelty’ does not apply to our work, as we explicitly argue the exact opposite. Our paper states, the core concept that data-driven, pre-specified adaptations to optimize trial efficiency predates Bradford Hill’s foundational 1948 randomized controlled trial.^11^

We appreciate Senn’s historical perspective that many “modern” adaptive methods are not new but have simply struggled for adoption. He draws a sobering parallel between the slow uptake of adaptive designs and the persistent, unscientific habit of dichotomization in clinical research. This reminds us that while clarity of definition is a necessary condition for progress, it is by no means a sufficient one. Progress also requires institutional memory—a conscious effort by the scientific community to retain historical lessons, both methodological and ethical, so that ‘modern’ adaptive designs do not continuously cycle through innovation and neglect.

## Re-examining ethical barriers

Begg characterizes response-adaptive randomization as “explicitly unethical.” His reasons recapitulate a view of clinical equipoise that is extremely common, but self-defeating and unworkable because it assumes equipoise requires a state of perfect, 50/50 uncertainty across the medical community.

This rigid definition fails in practice for three reasons. First, equipoise is consistent with no expert being uncertain because trials can have social value when they target disagreements between experts who have strong but conflicting treatment recommendations. Second, in reality, the expert community is rarely equally divided: an imbalance of opinion typically exists both at trial initiation and as data progressively emerge. Third, and more importantly, requiring an equal distribution of expert opinion or uncertainty would regard it as unethical to randomize participants to an intervention favored by a reasonable minority of the expert community, even if that minority was 40% of experts. But, if it is ethical for those experts to provide that intervention to patients with certainty in clinical practice, it cannot be unethical to allow participants to be randomized to that intervention with some positive probability.^12^

Despite its unfortunate name, equipoise does not require an equal distribution of anything.^13^ Freedman made this point 30 years ago.^14^ Because trial design decisions are inextricably scientific and ethical, identifying and deploying appropriate designs requires a rich engagement with both sets of issues. Just as Senn argues that the World Medical Association incorrectly conflates the methodological tool of a placebo with the unethical act of withholding treatment, statisticians and methodologists must not conflate adjusting randomization proportions with the unethical act of providing participants with a level of care that would not be recommended for them by even a reasonable minority of experts.

## Conclusion: Methodology and ethics as an integrated discipline

While centering the choice of trial design on ethics is not enough to solve these entrenched issues on its own, it is a necessary first step toward a system that better serves both the patient and the scientific community. Exposing these linguistic and ethical frictions is, as Senn’s comparison suggests, only the beginning of a long process toward methodological maturity and adoption in practice.

Crucially, this maturity requires us to view trial design and ethical evaluation not as two separate, consecutive steps, but as a single, integrated discipline. True ethical perspective must matter at the very moment design choices are being contemplated, embedding itself into every subsequent methodological step and data analysis during the conduct of the trial. When ethics is separated from methodology, it risks being reduced to a purely administrative exercise focused on checkboxes and procedures rather than the core integrity of the science.

This perspective recasts the historical debate surrounding value and waste in clinical development. When we invoke Doug Altman’s famous call for “less research, better research, and research done for the right reasons,”^15^ we do not argue for an absolute reduction in scientific inquiry. Rather, we advocate for a shift away from poorly designed, ethically unreflective trials that waste finite patient resources. In this light, we must push back against skepticism—such as Begg’s concerns regarding the “bandwidth” of the ethics community to evaluate technical design elements. The complexity of integrating sophisticated methodology with ethical oversight does not justify a suboptimal status quo; instead, it demands a more rigorous, collaborative framework for trial evaluation.
